# Monotherapy with darunavir/ritonavir is effective and safe in clinical practice

**DOI:** 10.7448/IAS.17.4.19813

**Published:** 2014-11-02

**Authors:** Juan Pasquau, Luis López-Cortés, Maria Isabel Mayorga, Pompeyo Viciana, María del Mar Arenas, Maria José Ríos, José Hernández-Quero, Manuel Castaño, María Dolores Merino, Manuel Márquez, Antonio Vergara, Alberto Terrón, Francisco Téllez, Carmen Hidalgo-Tenorio

**Affiliations:** 1 Infectious Diseases, Hospital Virgen de las Nieves, Granada, Spain; 2 Infectious Diseases, Hospital Virgen del Rocío, Sevilla, Spain; 3 Infectious Diseases, Hospital Carlos Haya, Málaga, Spain; 4 Infectious Diseases, Hospital Virgen de Macarena, Sevilla, Spain; 5 Infectious Diseases, Hospital Clínco San Cecilio, Granada, Spain; 6 Infectious Diseases, Complejo Hospitalario de Huelva, Huelva, Spain; 7 Infectious Diseases, Hospital Virgen de la Victoria, Málaga, Spain; 8 Infectious Diseases, Hospital de Puerto Real, Cádiz, Spain; 9 Infectious Diseases, Hospital de Jerez, Jerez, Spain; 0 Infectious Diseases, Hospital de La Línea, La Línea (Cádiz), Spain

## Abstract

**Introduction:**

Monotherapy against HIV has undoubted theoretical advantages and has good scientific fundaments. However, it is still controversial and here we will analyze the efficacy and safety of MT with darunavir with ritonavir (DRV/r) on patients who have received this treatment in our hospitals.

**Materials and Methods:**

Observational retrospective study that includes patients from 10 Andalusian hospitals that have received DRV/r in MT and that have been followed over a minimum of 12 months. We carried out a statistical descriptive analysis based on the profile of patients who had been prescribed MT and the efficacy and safety that were observed, paying special attention to treatment failure and virological evolution.

**Results:**

DRV/r was prescribed to 604 patients, of which 41.1% had a CD4 nadir <200/mmc. 33.1% had chronic hepatitis caused by HCV, had received an average of five lines of previous treatment and had a history of treatment failure to analogues in 33%, to non-analogues 22 and protease inhibitors (PI) in 19.5%. 76.6% proceeded from a previous treatment with PI. The simplification was the main criteria for the instauration of MT in the 81.5% and the adverse effects in the 18.5%. We managed to maintain MT in 84% of cases, with only 4.8% of virological failure (VF) with viral load (VL) >200 c/mL and 3.6% additional losses due to VF with VL between 50 and 200 copies/mL. Thirty three genotypes were performed after failure without findings of resistance mutations to DRV/r or other IPs. Only 23.7% of patients presented some blips during the period of exposition to MT. Eighty seven percent of all determinations of VL had <50 copies/mL, and only 4.99% had >200 copies/mL. Although up to 14.9% registered at some point an AE, only 2.6% abandoned MT because of AE and 1.2% because of voluntary decision. Although the average of total and LDL cholesterol increases 10 mg/dL after 2 years of follow-up, so did HDL cholesterol in 3mg/dL and the values of triglycerides (−14 mg/dL) and GPT (−6 UI/mL) decreased. The average count of CD4 lymphocytes increased from 642 to 714/mm^3^ at 24 weeks.

**Conclusions:**

In a very broad series of patients obtained from clinical practice, data from clinical trials was confirmed: MT with DRV as a de-escalation strategy is very safe, it's associated to a negligible rate of adverse effects and maintains a good suppression of HIV replication. VF (with >50 or >200 copies/mL) is always under 10% and in any case without consequences.

